# Using breath carbon monoxide to validate self-reported tobacco smoking in remote Australian Indigenous communities

**DOI:** 10.1186/1478-7954-8-2

**Published:** 2010-02-20

**Authors:** David J MacLaren, Katherine M Conigrave, Jan A Robertson, Rowena G Ivers, Sandra Eades, Alan R Clough

**Affiliations:** 1 School of Public Health, Tropical Medicine and Rehabilitation Sciences, James Cook University, Cairns, Queensland, Australia; 2 Drug Health Service, Royal Prince Alfred Hospital, Sydney, New South Wales, Australia; 3 Sydney Medical School, University of Sydney, New South Wales, Australia; 4 Illawarra Aboriginal Medical Service, Wollongong, New South Wales, Australia; 5 Baker IDI Heart and Diabetes Institute, Melbourne, Victoria, Australia; 6 School of Indigenous Australian Studies, James Cook University, Cairns, Queensland, Australia

## Abstract

**Background:**

This paper examines the specificity and sensitivity of a breath carbon monoxide (BCO) test and optimum BCO cutoff level for validating self-reported tobacco smoking in Indigenous Australians in Arnhem Land, Northern Territory (NT).

**Methods:**

In a sample of 400 people (≥16 years) interviewed about tobacco use in three communities, both self-reported smoking and BCO data were recorded for 309 study participants. Of these, 249 reported smoking tobacco within the preceding 24 hours, and 60 reported they had never smoked or had not smoked tobacco for ≥6 months. The sample was opportunistically recruited using quotas to reflect age and gender balances in the communities where the combined Indigenous populations comprised 1,104 males and 1,215 females (≥16 years). Local Indigenous research workers assisted researchers in interviewing participants and facilitating BCO tests using a portable hand-held analyzer.

**Results:**

A BCO cutoff of ≥7 parts per million (ppm) provided good agreement between self-report and BCO (96.0% sensitivity, 93.3% specificity). An alternative cutoff of ≥5 ppm increased sensitivity from 96.0% to 99.6% with no change in specificity (93.3%). With data for two self-reported nonsmokers who also reported that they smoked cannabis removed from the analysis, specificity increased to 96.6%.

**Conclusion:**

In these disadvantaged Indigenous populations, where data describing smoking are few, testing for BCO provides a practical, noninvasive, and immediate method to validate self-reported smoking. In further studies of tobacco smoking in these populations, cannabis use should be considered where self-reported nonsmokers show high BCO.

## Introduction

Over the past two decades, smoking rates in Australia have halved in the general population from 35% to 18% with predictions of 14% by 2020[1]. However, for Indigenous Australians, smoking rates appear to have remained unchanged. Nationally, 51% of Indigenous men and 47% of Indigenous women report to be regular smokers[2].Smoking rates vary across Indigenous Australian populations. For example, much higher rates of between 59% to 83%[3-8] have been documented in some remote communities of the Northern Territory (NT), with up to 92% of people reporting a history of tobacco use in one community[3].

Indigenous Australians experience a burden of disease 2.4 times that of non-Indigenous Australians[9], with the gap between Indigenous and non-Indigenous life expectancy at birth in 2009 estimated to be 11.5 years for men and 9.7 years for women[10]. About 12% of the burden of disease[9] and 20% of indigenous deaths[11] are attributable to tobacco use. In 2008, the Australian government made a commitment to "closing the gap" in Indigenous Australian life expectancy[12], including addressing smoking[13].

Documenting tobacco use in Indigenous communities has typically relied on self-report in surveys, with a few studies using biochemical markers to verify self-report. Urine cotinine has been used in both urban[14] and remote clinic-based studies[7]. However, this involves the complexities and cost of obtaining and testing a urine sample and does not provide an immediate assessment of smoking. Portable, hand-held Breath Carbon Monoxide (BCO) analyzers are tools used to immediately assess smoking status. They are suitable for both clinical and community-based studies[15-23] and are being used in a small number of Indigenous Australian settings[24-26]. The utility of BCO analyzers and optimum BCO cutoff to distinguish smokers and nonsmokers is being investigated in different populations around the world[27-36] with different cutoff levels recommended in different populations dependent on the intended use of the BCO test. These include: assessing antenatal smoking[15,16,36]; clinical or community surveys[17,22,27-29,32,33,37]; validating smoking cessation[18,20,30]; assessing passive smoking[17] or environmental pollution[35]; or investigating sociocultural patterns of smoking[23]. There is, however, no guidance for the optimum BCO cutoff level to validate self-reported tobacco smoking in community-based surveys in Indigenous Australian populations.

This paper examines the sensitivity and specificity of the BCO test and the optimal cutoff level to distinguish between smokers and nonsmokers in three remote Indigenous populations.

## Methods

### Setting

The study was conducted in three Arnhem Land communities with a combined Indigenous population of 3,770, including 1,104 males and 1,215 females aged ≥16 years. Contemporary life is strongly influenced by traditional social and cultural norms and practices, with more than 20 tribal groups and seven major language groups represented across the three communities[38]. English is a second or third language[39]. Tobacco was introduced in the 17^th ^century by Macassan traders from the Indonesian archipelago, and extended with the expansion of the pastoral industry and Christian missions during the early 20^th ^century[40].

### Sampling

Between July 2008 and February 2009, 400 Indigenous people (aged ≥16), comprising 15% of the targeted population, were interviewed in the baseline phase of a community-based intervention study.

Local community members were employed as research workers to assist in the recruitment of participants, to interpret when local language was required, and to assist with BCO testing. Given that random sampling is impractical and intrusive in these communities[41], participants were opportunistically invited to participate using quotas to reflect age and gender balances. Interviews occurred in public spaces or in people's homes.

### Community Survey: Self-reported tobacco smoking and BCO

Using a structured questionnaire, participants were asked about smoking status, smoking history, and pattern of tobacco use. Interviews were conducted by authors DM, JR, and AC, in most cases with local research workers. Participants were asked: "Do you smoke tobacco?" If the participant answered yes, a series of questions including the type of tobacco product used, the amount used, when/how the participant started smoking, and time since last cigarette were asked. Participants were also asked if they chewed tobacco, smoked tobacco in a pipe, or smoked "tailor made" and/or "roll your own: cigarettes.

Where required, research workers from the local community were able to provide a personal assessment of study participants' smoking status[41], a feasible indicator given sharing tobacco is an integral component of the collective social fabric of community life[42].

BCO was measured at interview using a hand-held Bedfont piCO+ Smokerlyzer (Bedfont Scientific, UK, http://www.bedfont.com). Participants were requested to inhale and hold their breath for 15 seconds before exhaling into the analyzer. A BCO cutoff of ≥7 ppm was used as recommended by the manufacturer. The BCO analyzers used in the study were calibrated by the manufacturer in May 2008 before the survey commenced and recalibrated by DM, according to the manufacturer's specifications, in November 2008. In trials of survey procedures, the acceptability of using a portable BCO analyzer with this population proved to be high. During the study, it was the experience of DM, JR, and AC that using the BCO analyser attracted participants into the study. The immediate return of BCO results provided an opportunity for participants to actively engage in discussion about tobacco smoking and have direct benefit from participating.

### Data analysis and approvals

Data were included in the analysis if: (i) self-reported smokers reported smoking tobacco within the preceding 24 hours; or (ii) self-reported nonsmokers reported never smoking tobacco or not smoking tobacco for ≥6 months[43]. Given the short half-life of BCO, data from self-reported occasional tobacco smokers who reported last smoking tobacco greater than 24 hours previously were not included in the analysis.

Self-reported smoking and BCO level were analyzed descriptively using sensitivity and specificity percentages and a Receiver Operating Characteristics (ROC) analysis. Sensitivity was defined as the proportion of all self-reported smokers for whom there was a positive BCO test, i.e., a BCO level at or above cutoff (≥7 ppm). Specificity was the proportion of nonsmokers for whom there was a negative BCO test, i.e., a BCO level below cutoff point (<7 ppm). Since a test should ideally have high sensitivity and high specificity, the average of sensitivity plus specificity was calculated for different cutoff points to find the highest level.

Ethics approval for the study was provided by the Human Research Ethics Committee of James Cook University (approval number H 3072) and the NT Department of Health and Families and Menzies School of Health Research (approval number 0707).

## Results

Among the 400 people interviewed, 300 (75%) reported they smoked tobacco, and 100 (25%) reported they did not. Four of the 400 interviewed explicitly refused a BCO test, and 19 were in such poor health that a BCO test would have been unnecessarily intrusive. BCO was not tested in a further 57 people interviewed primarily because they had no time to take the BCO test (n = 40) or because a BCO analyzer was not available at the time of interview (n = 17). The remaining 320 people who provided both a BCO test and information about their tobacco use included 260 who self-reported they smoked tobacco and 60 people who self-reported they did not smoke tobacco. Eleven of the 260 were occasional tobacco smokers and reported they had not smoked within the preceding 24 hours. In accordance with inclusion criteria, these 11 occasional smokers were not included in the analysis. Therefore, BCO tests for 249 self-reported smokers and 60 self-reported nonsmokers were analyzed. The proportions of self-reported smokers (81% = 249/309) and nonsmokers (19% = 60/309) in this subsample were similar to proportions of self-reported smokers (75%) and nonsmokers (25%) in the sample overall (|z| = 1.90, P = 0.057).

### Sensitivity and Specificity

Of the 249 self-reported smokers, 10 had BCO below the cutoff of ≥7 ppm (96.0% sensitivity), and of the 60 self-reported nonsmokers, four had BCO ≥7 ppm (93.3% specificity) (Table 1).

**Table 1:** Breath carbon monoxide (BCO) and self-reported smoking status

BCO level (p.p.m.)	Self-reported non-smokers (n)	Self-reported smokers (n) in BCO range				Total (n)
		**0-5 p.p.m.**	**6-10 p.p.m.**	**11-15 p.p.m.**	**>15 p.p.m.**	

1	22					22
2	20	**1**				21
3	11					11
4	3					3
5		**2**				2
6			**7**			7
≥7	**4**		42	66	131	243

Total						309

**Figure 1:**
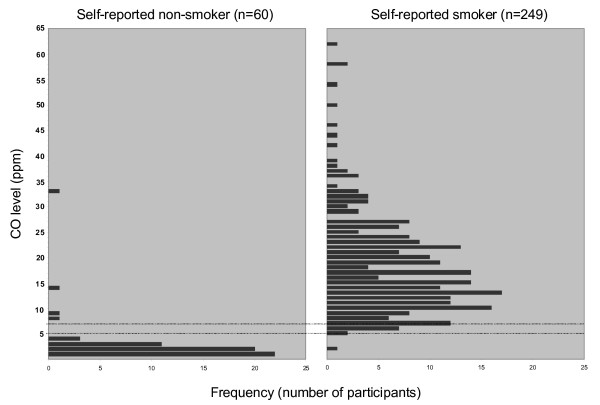
**Bar chart for breath carbon monoxide (BCO) measurements for self-reported nonsmokers and smokers**. The two lines indicate the cutoffs of ≥7 ppm and ≥5 ppm.

Of the four self-reported nonsmokers with BCO ≥7 ppm, three were males, two of whom stated they did not smoke tobacco but smoked cannabis (BCO 8 ppm and 33 ppm) (Figure 1). The third male (BCO 14 ppm) provided no comment, but local research workers later suggested that he smoked cannabis (Figure 1). One self-reported female nonsmoker with BCO ≥7 ppm (BCO 9 ppm, Figure 1) provided no further detail at interview, and local research workers were not present at interview to assist in clarifying the discrepancy.

Nine of the 10 self-reported smokers with BCO level <7 ppm provided information about time since last cigarette. Five reported their last cigarette was smoked approximately 8-12 hours prior (including three whose last cigarette was smoked the previous evening). Two had smoked approximately six hours prior, and two had their last cigarette within two hours before testing (data not shown).

Table 2 shows changes in sensitivity and specificity at different BCO cut-offs. The highest average for the combined sensitivity and specificity (96%) occurs at BCO cutoffs of ≥5 ppm and ≥6 ppm (Table 2).

**Table 2:** Sensitivity and specificity of various breath carbon monoxide (BCO) cutoff levels

CO cut-off ≥ (p.p.m.)	Sensitivity	Specificity	(Sensitivity + specificity)/2
1	1.000	0.000	0.50
2	1.000	0.367	0.68
3	0.996	0.700	0.85
4	0.996	0.883	0.94
5	0.996	0.933	**0.96**
6	0.988	0.933	**0.96**
7	0.960	0.933	0.95
8	0.912	0.933	0.92
9	0.888	0.950	0.92
10	0.855	0.967	0.91

### Alternative Cut-off Level

If a cutoff level of ≥5 ppm had been used in the study, the number of false negative tests in self-reported smokers would have been reduced by nine from 10 to 1, a reduction in the proportion of negative tests from 15% to 2%. Using a BCO cutoff of ≥ 5 ppm would substantially increase the sensitivity in the study from 96.0% to 99.6% with no change in specificity (93.3%) (Table 2). However excluding data for the two male cannabis users who said they did not smoke tobacco from analysis increased specificity to 96.6% (data not shown).

**Figure 2:**
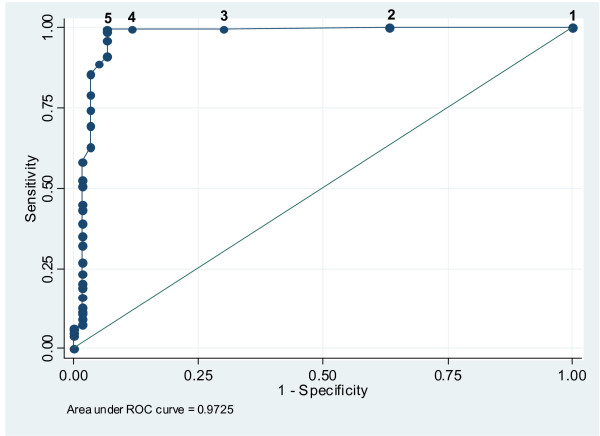
**For receiver operating characteristic (ROC) analysis, using data for all participants, 1-specificity (x-axis) was plotted against sensitivity at breath carbon monoxide (BCO) cutoff levels from 1 ppm to 10 ppm**. The numbers placed along the ROC curve indicate BCO cutoff levels.

An ROC analysis was performed to assess the diagnostic accuracy of BCO across the range of possible cutoff values (Figure 2). The significant contribution to the area under the curve (AUC = 0.972, P < 0.001) at a BCO cutoff of ≥5 ppm indicates the considerable power the BCO marker holds to discriminate between smokers and nonsmokers in this population. With the data for the two male self-reported cannabis smokers excluded from the ROC analysis, the area under the curve at a BCO cutoff of ≥5 ppm increased marginally to AUC = 0.989. For prevalence estimates in the sample, a cutoff level of ≥7 ppm would have estimated a prevalence of 79% of smokers. Using a cutoff level of ≥5 ppm would have estimated a prevalence of 82% of smokers. With data for the two male cannabis smokers excluded, using a cutoff level of ≥5 ppm would have estimated tobacco smoking prevalence of 81%, the proportion of self-reported smokers in the sample of 400 people interviewed in the study overall.

## Discussion

These findings indicate that BCO can be effectively used to validate self-reported smoking in remote Australian Indigenous communities. The strong agreement between self-reported smoking and BCO indicates that self-reported smoking can be considered a reliable measure in this population.

Limitations of the study include that the sample was not randomly selected and so results cannot be generalized. However, participants were recruited to reflect each community's age and gender characteristics. Although BCO was not tested in all participants, the gender composition and proportions of self-reported smokers among those who provided a BCO test were similar to the sample overall. Confidence in the results is further reinforced by the high level of agreement between BCO and self-report, by the similarly high smoking rates found in other studies in the region, and because community members themselves informed researchers that results reflected their own family and community experience.

The community-based survey method recorded self-reported smoking status and immediately returned BCO test results to study participants. This allowed discrepancies between self-report and BCO to be investigated at the time of interview with further questions about smoking history or pattern of use, which assisted in refining the data.

Ten (4%) self-reported smokers had BCO below the ≥7 ppm cutoff. In nine of these, the self-reported time since last cigarette was between 2 and 12 hours prior to BCO test. Three of these nine had not smoked since the previous evening. Given that BCO has a half-life of between 3 and 4 hours and can decline by 2.1 to 7.5 ppm per hour, depending on the initial BCO level[44], there was sufficient time for BCO to decline below the cutoff. Smokers consistently reported periods of heavy and light smoking with a greater amount of tobacco smoked in the first few days after fortnightly paydays. Similar to smoking patterns documented in other Indigenous communities[42], the majority of participants reported regularly running out and frequently requesting tobacco from family and friends. This provides a plausible explanation for self-reported tobacco smokers who only smoke occasionally and/or have low BCO. Although those who smoke few cigarettes per day can also have normal BCO[21,23,44], lapsed time since last cigarette, independent of the number of cigarettes smoked, accounted for most self-reported smokers with low BCO in this study.

The study also documented four (7%) self-reported nonsmokers with BCO ≥7 ppm. While such discrepancies may allude to false self-report or exposure to secondhand smoke, two of these four results could be accounted for by cannabis use. It is possible that exposure to secondhand smoke contributed to the small number of false positives in this study, however cannabis use in this population is likely to be an important factor. Studies of cannabis use in similar communities in the same region indicate that most cannabis users (94%) blend cannabis with tobacco and smoke the mixture in handmade "bucket bongs," with tobacco smokers about 19 times more likely than nonsmokers to also smoke cannabis[45]. Given that up to two-thirds of males and half of females regularly use cannabis in the region's communities[45,46], cannabis use should be considered where self-reported nonsmokers show high BCO in further self-reported smoking validation studies. The present study did not systematically collect data about cannabis use at interview. A study investigating both tobacco and cannabis smoking requires a different approach, including suitable protocols designed to minimize the ethical and legal risks of studying illegal behaviors in these disadvantaged and disempowered populations in Australia[45].

Only a few studies in Indigenous Australian populations have used biomarkers to verify self-reported smoking. A community-based study in an urban population recorded 10% of self-reported nonsmokers with BCO above cutoff of 9 ppm[26], while a clinic-based study in a remote population tested BCO but did not analyze discrepancies[25]. Two clinic-based studies using urine cotinine, one remote and one urban, both found higher discrepancies. The remote study found 15% of self-reported nonsmokers produced levels above cutoff (539 nmom/l)[7], while the urban study found 17% of self-reported nonsmoking pregnant women produced levels above cutoff (250 ng/ml)[14]. In the study reported here, 7% of self-reported nonsmokers had BCO above cutoff (≥7 ppm). Caution is, however, required before making direct comparison between these studies given the different population groups, recruitment methods, sample size, biomarkers, and study context.

A range of BCO cutoff levels has been used to validate self-reported smoking in different population groups around the world. Cutoffs as high as 10 ppm have been used[20,47,48]. Others have used 9 ppm[22,26,37,44]; 8 ppm[19]; 7 ppm[28]; 6.5 ppm[17]; or 6 ppm[21]. Several studies recommend cutoffs as low as 2 to 3 ppm[29,31,36]. However, self-reported smoking status and BCO level can vary between ethnic groups in the same location[23]. This suggests possible cultural or communication differences in responses to questions about smoking, a challenge well-known in remote Aboriginal communities[7,39]. Different sociocultural patterns of smoking may mean different BCO cutoffs are required in different populations. In this study population, reducing the BCO cutoff from ≥7 ppm to ≥5 ppm would increase the self-reported smokers verified from 96.0% to 99.6%, while self-reported nonsmokers verified would remain unchanged at 93.3%. With better information in future studies about those who smoke cannabis and not tobacco in these communities, the proportion of self-reported nonsmokers of tobacco verified could be as high as 96.6%.

## Competing interests

The authors declare that they have no competing interests.

## Authors' contributions

DM participated in collection, analysis, and interpretation of data, and drafted and edited the manuscript. KC made substantial contributions to the conception and design of the study, analyzed and interpreted data, and revised the manuscript. JR participated in collection of data and revised the manuscript. RI participated in the conception and design of the study and revised the manuscript. SE participated in the conception and design of the study and revised the manuscript. AC led the conception and design of the study, participated in data collection, led the statistical analysis of results, and critically revised all sections for intellectual content. All authors read and approved the final manuscript.

## References

[B1] Gartner CE Barendregt JJ Hall WD Predicting the future prevalence of cigarette smoking in Australia: how low can we go and by when? Tob Control 2009 tc.2008.027615 19179370 10.1136/tc.2008.027615

[B2] Trewin D National Aboriginal and Torres Strait Islander Health Survey. Australia 2004-05 2006 Canberra: Australian Bureau of Statistics

[B3] Clough A Robertson J MacLaren D The gap in tobacco use between remote Indigenous Australian communities and the Australian population can be closed Tob Control 2009 18 335 336 10.1136/tc.2009.030577 19633148

[B4] Clough AR Guyula T Yunupingu M Burns CB Diversity of substance use in eastern Arnhem Land (Australia): patterns and recent changes Drug Alcohol Rev 2002 21 349 356 10.1080/0959523021000023207 12537704

[B5] Hoy WE Norman RJ Hayhurst BG Pugsley DJ A health profile of adults in a Northern Territory Aboriginal community, with an emphasis on preventable morbidities Aust NZ J Public Health 1997 21 121 126 10.1111/j.1467-842X.1997.tb01670.x 9161065

[B6] Ivers R Castro A Parfitt D Bailie R D'Abbs P Richmond R Evaluation of a multi-component community tobacco intervention in three remote Australian Aboriginal communities Aust N Z J Public Health 2006 30 132 136 10.1111/j.1467-842X.2006.tb00105.x 16681333

[B7] McDonald SP Maguire GP Hoy WE Validation of self-reported cigarette smoking in a remote Australian Aboriginal community Aust N Z J Public Health 2003 27 57 60 10.1111/j.1467-842X.2003.tb00380.x 14705268

[B8] Watson C Fleming J Alexander K A survey of drug use patterns in Northern Territory Aboriginal communities: 1986-1987 1988 Darwin: Northern Territory Department of Health and Community Services, Drug and Alcohol Bureau

[B9] Vos T Barker B Begg S Stanley L Lopez AD Burden of disease and injury in Aboriginal and Torres Strait Islander Peoples: the Indigenous health gap Int J Epidemiol 2009 38 470 477 10.1093/ije/dyn240 19047078

[B10] New estimates of Indigenous life expectancy released : ABS http://www.abs.gov.au/AUSSTATS/abs@.nsf/0/C65F4C150DD0497ACA2575BE002656BC?OpenDocument

[B11] Vos T Barker B Stanley L Lopez A The burden of disease and injury in Aboriginal and Torres Strait Islander peoples 2003 2007 Brisbane: School of Population Health, The University of Queensland

[B12] Department of Families Housing Community Services and Indigenous Affairs Closing the Gap on Indigenous Disadvantage: the challenge for Australia 2009 Canberra Commonwealth of Australia

[B13] Rudd K Joint Media Release with the Minister for Health and Ageing and the Minister for Indigenous Affairs - Rudd Government Tackles Indigenous Smoking Rates and Health Workforce in next Down Payments on Closing the Gap 2008 Australia PMo ed. Canberra Commonwealth of Australia

[B14] Gilligan C Sanson-Fisher R Eades S Wenitong M Panaretto K D'Este C Assessing the accuracy of self-reported smoking status and impact of passive smoke exposure among pregnant Aboriginal and Torres Strait Islander women using cotinine biochemical validation Drug Alcohol Rev 2009 9999 10.1111/j.1465-3362.2009.00078.x 20078680

[B15] Campbell E Sanson-Fisher R Walsh R Smoking status in pregnant women: Assessment of self-report against carbon monoxide (CO) Addict Behav 2001 26 1 9 10.1016/S0306-4603(00)00070-8 11196282

[B16] Campbell E Walsh RA Sanson-Fisher R Burrows S Stojanovski E A group randomised trial of two methods for disseminating a smoking cessation programme to public antenatal clinics: effects on patient outcomes Tob Control 2006 15 97 102 10.1136/tc.2004.011049 16565456 PMC2563553

[B17] Deveci SE Deveci F Acik Y Ozan AT The measurement of exhaled carbon monoxide in healthy smokers and non-smokers Respir Med 2004 98 551 556 10.1016/j.rmed.2003.11.018 15191041

[B18] Hung J Lin CH Wang JD Chan CC Exhaled carbon monoxide level as an indicator of cigarette consumption in a workplace cessation program in Taiwan J Formos Med Assoc 2006 105 210 213 10.1016/S0929-6646(09)60307-7 16520836

[B19] Jarvis MJ Tunstall-Pedoe H Feyerabend C Vesey C Saloojee Y Comparison of tests used to distinguish smokers from nonsmokers Am J Public Health 1987 77 1435 1438 10.2105/AJPH.77.11.1435 3661797 PMC1647100

[B20] Jatlow P Toll BA Leary V Krishnan-Sarin S O'Malley SS Comparison of expired carbon monoxide and plasma cotinine as markers of cigarette abstinence Drug Alcohol Depend 2008 98 203 209 10.1016/j.drugalcdep.2008.05.013 18650033 PMC2577604

[B21] Middleton ET Morice AH Breath carbon monoxide as an indication of smoking habit Chest 2000 117 758 763 10.1378/chest.117.3.758 10713003

[B22] Morabia A Bernstein MS Curtin F Berode M Validation of Self-Reported Smoking Status by Simultaneous Measurement of Carbon Monoxide and Salivary Thiocyanate Prev Med 2001 32 82 88 10.1006/pmed.2000.0779 11162330

[B23] Pearce MS Hayes L Newcastle Heart P, Newcastle Thousand Families S Self-reported smoking status and exhaled carbon monoxide: results from two population-based epidemiologic studies in the North of England Chest 2005 128 1233 1238 10.1378/chest.128.3.1233 16162711

[B24] Adams K Briggs V Galnya Angin (Good Air) Partnerships in Indigenous Tobacco Control 2005 Melbourne: Centre for Excellence in Tobacco Control

[B25] Ivers RG Farrington M Burns CB Bailie RS D'Abbs PH Richmond RL Tipiloura E A study of the use of free nicotine patches by Indigenous people Aust N Z J Public Health 2003 27 486 490 10.1111/j.1467-842X.2003.tb00819.x 14651391

[B26] Perkins J Sanson-Fisher R Blunden S Lunnay D Redman S Hensley M The prevalence of drug use in urban Aboriginal communities Addiction 1994 89 1319 1331 10.1111/j.1360-0443.1994.tb03311.x 7804093

[B27] Chatkin J Fritscher L de Abreu C Cavalet-Blanco D Chatkin G Wagner M Fritscher C Exhaled carbon monoxide as a marker for evaluating smoking abstinence in a Brazilian population sample Prim Care Respir J 2007 16 36 40 10.3132/pcrj.2007.00008 17297525 PMC6634175

[B28] Chatrchaiwiwatana S Ratanasiri A Exhaled carbon monoxide level and smoking status in urban Khon Kaen adults J Med Assoc Thai 2008 91 1669 1676 19127787

[B29] Cropsey KL Eldridge GD Weaver MF Villalobos GC Stitzer ML Expired Carbon Monoxide Levels in Self-Reported Smokers and Nonsmokers in Prison Nicotine Tob Res 2006 8 653 659 10.1080/14622200600789684 17008192

[B30] Holt S Timu-Parata C Ryder-Lewis S Weatherall M Beasley R Efficacy of bupropion in the indigenous Maori population in New Zealand Thorax 2005 60 120 123 10.1136/thx.2004.030239 15681499 PMC1747282

[B31] Javors M Hatch J Lamb R Cut-off levels for breath carbon monoxide as a marker for cigarette smoking Addiction 2005 100 159 167 10.1111/j.1360-0443.2004.00957.x 15679745

[B32] Kentala J Utriainen P Pahkala K Mattila K Verification of adolescent self-reported smoking Addict Behav 2004 29 405 411 10.1016/j.addbeh.2003.08.012 14732430

[B33] Kunze U Böhm G Ferstl F Groman E Assessing smoking behaviour among medical students by the measurement of expired carbon monoxide (CO) WMW Wiener Medizinische Wochenschrift 2009 159 14 16 10.1007/s10354-008-0635-7 19225730

[B34] Lamb RJ Morral AR Kirby KC Iguchi MY Galbicka G Shaping smoking cessation using percentile schedules Drug Alcohol Depend 2004 76 247 259 10.1016/j.drugalcdep.2004.05.008 15561476

[B35] Shafiq M Khan S Khawaja MR Haque S Khan JA Socio-demographic correlates of exhaled breath carbon monoxide in Karachi's adult population J Pak Med Assoc 2008 58 75 78 18333525

[B36] Usmani Z Craig P Shipton D Tappin D Comparison of CO breath testing and women's self-reporting of smoking behaviour for identifying smoking during pregnancy Subst Abuse Treat Prev Policy 2008 3 4 10.1186/1747-597X-3-4

[B37] Jagoe K Edwards R Mugusi F Whiting D Unwin N Tobacco smoking in Tanzania, East Africa: population based smoking prevalence using expired alveolar carbon monoxide as a validation tool Tob Control 2002 11 210 214 10.1136/tc.11.3.210 12198270 PMC1759028

[B38] Tindale N Aboriginal tribes of Australia: their terrain environmental controls distribution limits and proper names 1974 Canberra: Australian National University Press

[B39] Trudgen RI Why warriors lie down and die: Djambatj mala towards an understanding of why Aboriginal people of Arnhem Land face the greatest crisis in health and education since European contact 2000 Darwin: Aboriginal Resource and Development Services

[B40] Brady M Historical and cultural roots of tobacco use among Aboriginal and Torres Strait Islander people Aust NZ J Publ Health 2002 26 120 124 10.1111/j.1467-842X.2002.tb00903.x 12054329

[B41] Clough AR Cairney S D'abbs P Parker R Maruff P Gray D O'Reilly B Measuring Exposure to Cannabis use and other Substance use in Remote Aboriginal Populations in Northern Australia: Evaluation of A 'Community Epidemiology' Approach using Proxy Respondents Addict Res Theory 2004 12 261 274 10.1080/16066350410001667143

[B42] Johnston V Thomas DP Smoking behaviours in a remote Australian Indigenous community: The influence of family and other factors Soc Sci Med 2008 67 1708 1716 10.1016/j.socscimed.2008.09.016 18938006

[B43] West R Hajek P Stead L Stapleton J Outcome criteria in smoking cessation trials: proposal for a common standard Addiction 2005 100 299 303 10.1111/j.1360-0443.2004.00995.x 15733243

[B44] Leitch DN Harkawat R Askew J Masel P Hendrick DJ Relation of expired carbon monoxide to smoking history, lapsed time, TLCO measurement and passive smoking Respir Med 2005 99 32 38 10.1016/j.rmed.2004.03.027 15672846

[B45] Clough AR D'Abbs P Cairney S Gray D Maruff P Parker R O'Reilly B Emerging patterns of cannabis and other substance use in Aboriginal communities in Arnhem Land, Northern Territory: a study of two communities Drug Alcohol Rev 2004 23 381 390 10.1080/09595230412331324509 15763742

[B46] Lee KS Clough AR Conigrave KM High levels of cannabis use persist in Aboriginal communities in Arnhem Land, Northern Territory Med J Aust 2007 187 594 595 18021054 10.5694/j.1326-5377.2007.tb01428.x

[B47] Jorenby DE Smith SS Fiore MC Hurt RD Offord KP Croghan IT Hays JT Lewis SF Baker TB Varying nicotine patch dose and type of smoking cessation counseling JAMA 1995 274 1347 1352 10.1001/jama.274.17.1347 7563558

[B48] Tonnesen P Norregaard J Mikkelsen K Jorgensen S Nilsson F A double-blind trial of a nicotine inhaler for smoking cessation JAMA 1993 269 1268 1271 10.1001/jama.269.10.1268 8437304

